# Supplementary material for: Species-Level Differences in Osmoprotectants and Antioxidants Contribute to Stress Tolerance of Quercus robur L., and Q. cerris L. Seedlings under Water Deficit and High Temperatures
Source: Plants (Basel). 2022 Jun 30;11(13):1744. doi: 10.3390/plants11131744 (PMC9269681; doi:10.3390/plants11131744)
Supplement: Supplementary file 1 [file plants-11-01744-s001.zip › plants-1772348-supplementary.pdf]

Table S1. Results of two-way ANOVA for the effects of Species (*Q. robur* and *Q. cerris*), Treatment (control, soil water withholding, and high air temperatures), and their interaction (Species*Treatment) on the listed parameters measured at leaf level. Significance values are indicated as: ns, non-significant P>0.05; *, P≤0.05; **, P≤0.01; ***, P≤0.001.

	Parameters	Species			Treatment			Species*Treatment		
		F		Sum of square	*F*		Sum of square	*F*		Sum of square
**Osmolytes**	**Proline (PRO)**	*12.71*	**	211.13	*22.32*	***	741.62	*0,92*	ns	30.45
	**Glycine betaine (GB)**	*4773.88*	***	6548.80	*1534.48*	***	4210	*818.61*	***	2245.9
	**Dimethylsulphoniopropionate (DMSP)**	*3018140*	***	1.77	*1990396*	***	2.33	*1229464*	***	1.44
**Antioxidant Defense Systems**	**Lipid peroxidation (MDA)**	*35.38*	***	104.4	*249.20*	***	1467.05	*3.63*	ns	21.37
	**Total non-protein thiols (GSH)**	*2.04*	ns	0.24	*69.29*	***	16.50	*15.03*	***	3.58
	**ABTS**	*40.47*	***	56.53	*29.62*	***	82.75	*7.72*	**	21.57
	**DPPH**	*63.91*	***	2.58	*65.70*	***	5.30	*62.67*	***	5.05
	**FRAP**	*0.01*	ns	0.01	*13.12*	***	36.53	*21.34*	***	59.42
	**Total phenolic content (TPC)**	*12.20*	**	0.14	*39.92*	***	0.93	*51.39*	***	1.19
	**Total flavonoid content (TFC)**	*29.36*	***	7.04	*6.59*	*	3.16	*0.18*	ns	0.08
	**Condensed tannins (CT)**	*417.06*	***	12.94	*276.68*	***	17.16	*96.09*	***	5.96
**Hormones**	**Indol-3-acetic acid (IAA)**	*10.32*	**	1206.9	*31.90*	***	7458.2	*11.00*	**	2572.7
	**Abscisic acid (ABA)**	*16.12*	**	18099	*69.53*	***	156167	*66.68*	***	149780
**Mineral elements**	**Nitrogen (N)**	*5607.1*	***	0.32	*1260.7*	***	0.14	*2756.0*	***	0.31
	**Sulphur (S)**	*2.03*	ns	0.00	*9.97*	**	0.00	*9.52*	**	0.00

Table S2. Leaf total Nitrogen (N) and Sulphur (S) content in leaves (% dry weight; mean±standard deviation) of *Q. robur* and *Q. cerris* seedlings. See the legend of Figure 1 for abbreviations of the treatments. Different small letters indicate significant differences across the different treatments for each species. Asterisks indicate significant differences between the two oak species within each treatment, Tukey's honestly significant difference (HSD) post hoc test (P≤0.05).

Species	Treatment	Nitrogen (N) concentration (%)	Sulphur (S) Concentration (%)
	**C**	2.56±0.01a*	0.25±0.01a*
Q. robur	**D**	2.42±0.01b*	0.22±0.01b
	**HS**	2.04±0.01d*	0.19±0.01c
Q. cerris	**C**	2.01±0.01d	0.22±0.01a
	**D**	2.09±0.01c	0.23±0.01a
	**HS**	2.13±0.01b	0.21±0.02a

Table S3. Results of the two-way ANOVA (*F* and *P* values) for the first and second principal components (PC1 and PC2, respectively) were extracted. The main effect of Species, Treatment, and the first-order interaction (*Species* x *Treatment*) are shown. The factor effect was considered significant at *P*<0.05.

Response variable	Factors	Degree of freedom	*F*-value	*P*-value
	Species	1	107.27	2.45E-07
**PC1**	*Treatment*	2	60.66	5.32E-07
	SpeciesxTreatment	2	33.24	1.28E-05
	Species	1	7.34	0.019
**PC2**	*Treatment*	2	328.91	3.31E-11
	SpeciesxTreatment	2	72.25	2.03E-07
